# Breed-typical front limb angular deformity is associated with clinical findings in three chondrodysplastic dog breeds

**DOI:** 10.3389/fvets.2022.1099903

**Published:** 2023-01-17

**Authors:** Anu K. Lappalainen, Hanna S. M. Pulkkinen, Sari Mölsä, Jouni Junnila, Heli K. Hyytiäinen, Outi Laitinen-Vapaavuori

**Affiliations:** ^1^Department of Equine and Small Animal Medicine, Faculty of Veterinary Medicine, University of Helsinki, Helsinki, Finland; ^2^EstiMates Oy, Turku, Finland

**Keywords:** angular deformity, dog, chondrodysplasia, conformation, range of motion, carpal valgus, external rotation, lameness

## Abstract

Angular front limb deformity (ALD) refers to an excessively curved limb conformation, which is seen in some chondrodysplastic dog breeds. Common characteristics of ALD include carpal valgus (VALG), front limb rotation (ROT), elbow incongruity, and lateral radial head subluxation. These may cause lameness and discomfort in affected dogs. The clinical impact and breed-specific characteristics of front limb conformation in chondrodysplastic breeds are unknown. This prospective and cross-sectional study aimed to investigate differences in front limb conformation between three chondrodysplastic breeds. We further evaluate whether front limb conformation is associated with clinical findings and limb function. We propose novel methods to classify findings in the interosseous space and to quantify lateral radial head subluxation. Data from a total of 224 front limbs from 112 dogs of three chondrodysplastic dog breeds (30 Standard Dachshunds, 29 Skye terriers, and 53 Glen of Imaal terriers) were included in the study. Front limb VALG and ROT were measured with a goniometer. From the radiographs, the elbow joint was graded for incongruity (INC), and the humeroradial angle (HRA) was measured to assess lateral radial subluxation. The association of front limb conformation with clinical signs and limb function was investigated using orthopedic examination, goniometric and kinetic measurements, and radiography. The breeds differed significantly in their front limb conformation. The Dachshund had the least ROT and the least radial head subluxation. The Skye terrier had the most VALG, the most radial head subluxation, and the largest prevalence of moderate and severe INC. The Glen of Imaal terrier had the most ROT. In addition, INC, ROT, VALG, and HRA were found to be independent of each other and were associated with several measurable clinical abnormalities and limb function such as pain, lameness, limited range of motion, and elbow joint osteoarthritis. This implies that VALG, ROT, and HRA could be used in addition to INC grading when choosing musculoskeletal characteristics of dogs suitable for breeding.

## 1. Introduction

Hansen (1) introduced the term chondrodystrophy to refer to dogs with a characteristic short-limbed appearance, which we now know is caused by the *fgf4* retrogene in chromosome 18 (2). As the recent genetic literature uses the term chondrodysplasia when referring to this breed-defining mutation (2, 3), the term chondrodysplasia will also be used in this paper in reference to short-limbed breeds. In chondrodysplastic dogs, the markedly curved conformation of the front limbs could also be called angular limb deformity (ALD) (4–7).

In chondrodysplastic dog breeds, ALD, caused by premature closure of the distal ulnar growth plate, is considered to be an inherited trait at least in Skye terriers (4) and Basset hounds (8). It leads to asymmetry in the length of the two parallel bones of the antebrachium: the radius and the ulna. To accommodate its growth in the space limited by the short ulna, the radius can become curved in both the mediolateral and craniocaudal planes (5, 9, 10). This may be accompanied by radial torsion and/or valgus position of the carpus. At the proximal end of the antebrachial bones, the radial head can sometimes become subluxated (5, 9, 10), which is considered a subjective finding with no method to quantify the amount of subluxation. Chondrodysplastic dog breeds can differ markedly in their appearance, but no information exists on how they differ in front limb conformation. Whether individual characteristics of ALD, such as carpal valgus, front limb rotation, or elbow incongruity and subluxation, occur together or present independently of each other is unknown.

Extreme conformational traits may be related to compromised function. Pain during flexion of the carpus and manipulation of the elbow joint has been reported in association with ALD, and passive extension of the elbow joint may also be limited (4, 7, 11). During weight bearing the dogs may hold the elbow in abduction and some dogs may show weight bearing concentrated on the medial side of the paw or plantigrade stance (4, 7). Lameness has been reported in affected dogs (4, 6). In addition to lameness, the deformity has been stated to cause other changes in the kinematics of the limbs, described as a lateral swinging motion of the elbow while walking, whereas the flexion and extension mainly take place at the shoulder joint (4, 7). Kinetic analyses can be used to study the locomotion of dogs (12–14). Unfortunately, only scarce information exists of locomotion of chondrodysplastic dogs or dogs with ALD (12).

Radiography and computed tomography are commonly used when examining dogs with premature closure of the distal ulnar growth plate and ALD. When investigating an adult dog, the growth plates will have already mineralized, and detection of a growth plate disorder relies on assessing the presence of the consequent ALD (4, 5, 7, 9, 11, 15). Additional radiographic findings include elbow joint incongruity and radiocarpal subluxation with remodeling of the carpal bones (4, 7, 9, 11). To assess elbow incongruity, a grading system (INC) has been developed by Lappalainen et al. (6) and validated by Pulkkinen et al. (16). This grading is currently used when screening chondrodysplastic dogs for elbow incongruity in Finland, with the aim of reducing the prevalence of elbow incongruity in these breeds by screening the dogs for the disease and assessing their suitability for breeding (6). Furthermore, an interesting radiographic finding of interosseous bone formation in the space between the radius and ulna has been reported using descriptions such as interosseous synostosis, epiphyseal new bone formation, and calcified plaques parallel to the distal ulna (4, 7, 9, 11). The etiology and significance of this finding remain elusive.

The aim of this study was to investigate differences in the conformation of the front limb between three chondrodysplastic dog breeds: Skye terrier, Glen of Imaal terrier, and Dachshund. Additionally, we evaluated whether limb conformation is associated with clinical findings and limb function. Also, we aimed to develop methods to classify findings in the interosseous space and to quantify lateral radial head subluxation. We hypothesized that the front limb conformation differs between the breeds and that an association exists between limb conformation and function.

## 2. Materials and methods

This study was approved by the National Animal Experiment Board in Finland (ESAVI/9184/04.10.07/2014) and the University of Helsinki Viikki Campus Research Ethics Committee (Statement 2/2017). All dog owners provided informed consent before participation in the study.

### 2.1. Recruitment and inclusion criteria

This was a prospective cross-sectional observational study. The Glen of Imaal terrier breed was selected due to breeders' concerns about the front limb conformation in the breed. Skye terrier was chosen as it has been previously studied by our research group, and Dachshunds were selected because worldwide it is the most common chondrodysplastic breed. The study was advertised through the social media, and dogs and dog owners were recruited through the respective breed clubs in the order of enrollment. The inclusion criteria were dog's age 1–10 years and registration in the Finnish Kennel Club breed registry. Exclusion criteria were history of orthopedic surgery and any health condition increasing the risks involved in sedation.

All dogs were examined at the University of Helsinki Veterinary Teaching Hospital during 2015–2017. Before the study, the owners filled in a questionnaire on thoracic limb lameness (6).

### 2.2. Orthopedic examination

After a physical examination (HP), an ECVS-certified veterinary surgeon (SM) performed an orthopedic examination. This included a lameness evaluation and palpation of the thoracic and pelvic limbs and spine as well as evaluation of conscious proprioception and withdrawal reflex. The spine was evaluated for pain and the extremities for pain, crepitation, swelling, decreased range of motion, and instability (yes/no).

### 2.3. Conformational and passive range of motion measurements

Conformational measurements were performed by a veterinarian (HP) or a veterinary physiotherapist (HH). For these measurements, the dog stood on a non-slippery surface, with the handler holding the dog in a straight and square standing position. The handler was instructed not to support the dog's weight or to let the dog lean on the handler's hands. The measurements were taken once with the tape measure and included the height at the withers (with the help of a spirit level placed at the highest point of the dorsal border of the scapula), the distance from the ground to the top of the olecranon (17), and the chest circumference behind the scapulae. Distal limb external rotation (ROT) and carpal valgus (VALG) were measured three times during weight bearing with the dog in a standing position using a universal goniometer according to a protocol described earlier (18), and the mean of the measurements was used in the statistical analyses. Passive range of motion (PROM) of the carpus, elbow, and shoulder was measured three times with the dog in lateral recumbency by using a universal goniometer as previously described (19), and the mean of the measurements was used in the statistical analyses.

### 2.4. Static weight bearing and temporospatial gait analysis

Locomotion-related measurements were performed by a veterinarian (HP) or a veterinary physiotherapist (HH). Static weight bearing as percentual weight distribution was measured with a pressure-sensitive platform (Stance Analyzer, Petsafe, Knoxville, TN, USA) (20). The equipment was calibrated with a known weight prior to each measurement session. The owner was positioned directly in front of the dog while the dog was standing in a square position on the analyzer with one foot on each quadrant of the measuring platform (21). A minimum of 10 measurements was recorded and saved, and the mean of the measurements was used in the statistical analyses.

Gait analysis was performed using a pressure-sensitive walkway (22) of width 90 cm and length 7 m, with a measurement frequency of 240 Hz (GAITRite Electronic Walkway, CIR Systems Inc., Peekskill, NY, USA). The handler changed sides between each trial to ensure a minimal effect on gait symmetry (23). Parameters recorded for the study included step length in centimeters, stance time in seconds, and total pressure index during trot. Several trials were performed in order to collect a minimum of 20 gait cycles for each dog, and the mean of the cycles' values for each parameter was used. Data were recorded and analyzed using the accompanying software (GaitFour, version 40f CIR Systems Inc., Havertown, PA, USA).

### 2.5. Radiographic examination and assessment of radiographs

The dogs were sedated for the radiographs with dexmedetomidine (0.1 mg/kg) and butorphanol (0.1 mg/kg) intramuscularly. The images were acquired using computed radiography with an automatic exposure detector, imaging plates, and a reader (Fujifilm FCR XG-1 CR-IR 346RU, Fuji Photo Film Co. Ltd., Tokyo, Japan). S-values of 100–300 were targeted to ensure image quality. The mediolateral (ML) antebrachial radiographs were acquired using the imaging protocol introduced by Lappalainen et al. (6). The craniocaudal (CrCd) images were obtained with the x-ray beam centered to mid-radius so that the whole antebrachium including the carpal joint was visible in the image. The elbow joint was positioned in a CrCd or ML position, but the wrist was allowed to remain in an anatomically rotated position. Mechanical restraints, such as foam wedges, sandbags, and tape, were used to aid in positioning the limb for the radiographs. The radiographs were assessed with OsiriX MD 9.0 (Pixmeo, Switzerland) or Horos DICOM viewer v. 2.1.1 (Horos Project).

**Figure 1:**
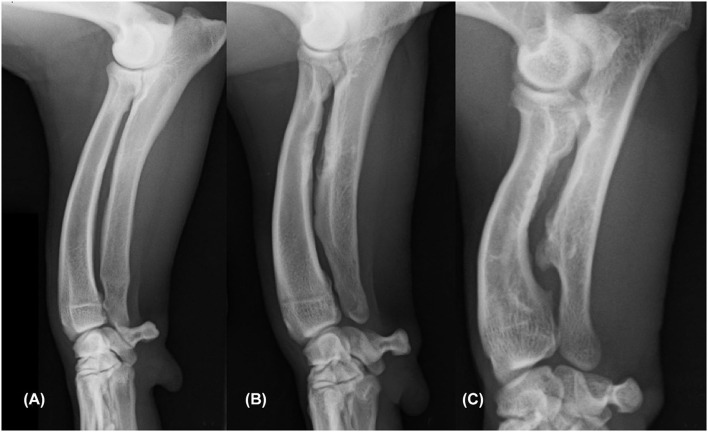
Mediolateral radiograph of the antebrachium showing an example of each of the three categories of cranial ulnar cortical remodeling (CUCR). The ulnar cortical findings were graded as CUCR0 **(A)**, CUCR1 **(B)**, and CUCR2 **(C)**.

The ML antebrachial radiographs were assessed for elbow joint incongruity, elbow joint osteoarthritis, and cranial ulnar cortical remodeling (CUCR). The incongruity grade (INC) was determined as the mode of altogether nine measurements by three investigators. These measurements were initially performed for a repeatability study by Pulkkinen et al. (16), which did not report the original INC measurements. A previously established grading system (INC0–INC3) was used (6). The presence of elbow joint osteoarthritis was assessed by AL according to International Elbow Working Group guidelines (24). Additionally, the presence of CUCR on a scale of 0 (none) to 2 (severe) (Table 1; Figure 1) was evaluated by HP.

**Table 1:** Grading matrix for cranial ulnar cortical remodeling (CUCR).

**Severity of cranial ulnar cortical new bone formation**	**Definition**
CUCR0	All of the following or only mild changes in one of the following: - No disruption in cortical definition - The cortex appears smooth/homogeneous - No bulging or new bone formation on the ulnar cortex
CUCR1	Moderate-severe changes in one or changes in more than one of the following: - The cortex is not well-defined - The cortex appears rough/heterogeneous - New bone formation is evident on the ulnar cortex
CUCR2	Ulnar physeal bulging near the distal ulnar growth plate with the bulge protruding distally

**Figure 2:**
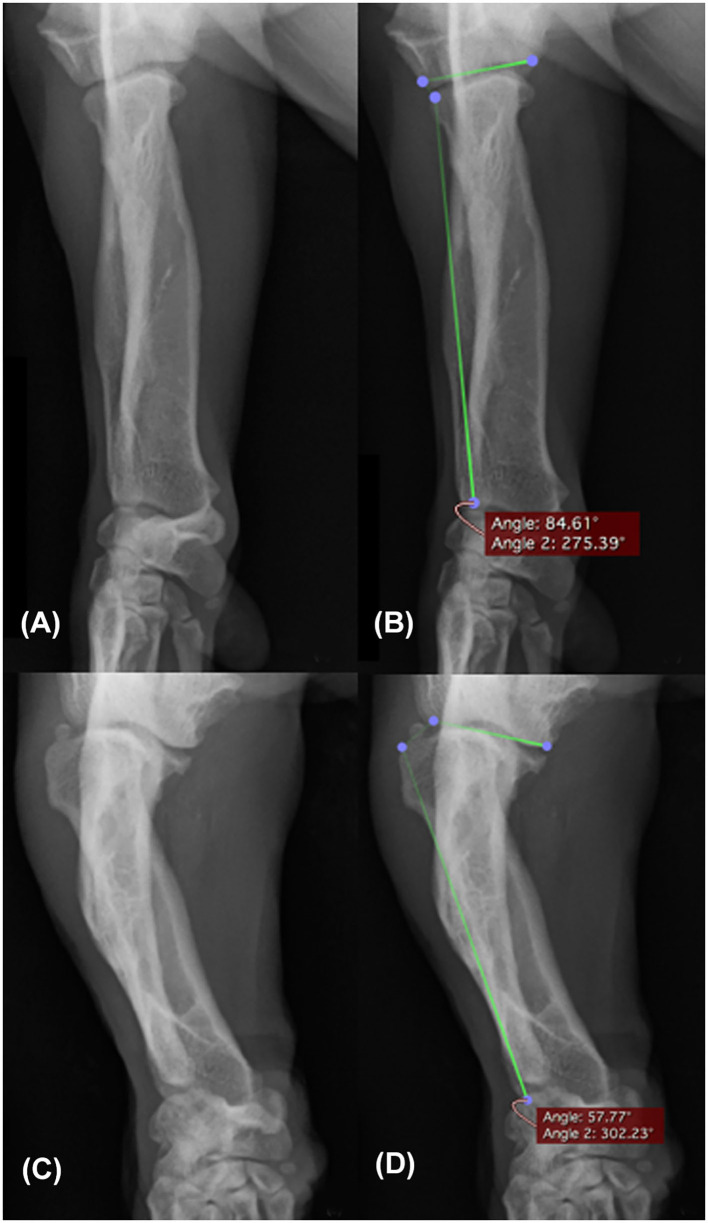
Craniocaudal radiograph of the antebrachium showing two examples of measuring the humeroradial angle. An elbow with subjectively no radial head luxation **(A)** measured 84.61° for the humeroradial angle **(B)**. An elbow with subjectively marked radial head luxation **(C)** measured 57.77° for the humeroradial angle **(D)**.

As a measure of lateral radial subluxation, the CrCd antebrachial radiographs were assessed by HP for the angle between the radial axis and the humeral joint surface (humeroradial angle, HRA). This was performed as a single measurement by using the angle measurement tool available in the radiograph viewing software. An example of measuring the HRA is shown in Figure 2. The first line was drawn parallel to the humeral joint surface. The second line was drawn parallel to the lateral axis of the radius, which was represented by a line from the lateral aspect of the radial head to the lateral aspect of the styloid process of the radius. The angle between these lines was then automatically determined by the imaging software.

### 2.6. Statistical analysis

The variables chosen to represent front limb conformation were ROT, VALG, HRA, and INC. To assess the effect of breed on these conformational variables, linear mixed effect models (ROT, VALG, HRA) and mixed cumulative logit models (INC) with the breed and limb as fixed terms and dog as the random subject effect were used. The conformational variables were investigated for associations between each other with scatter plots and regression lines and with Spearman correlation coefficients.

To investigate the associations between the conformational variables and clinical variables, several models were used as required by the data, and each conformational and clinical variable was evaluated separately. ROT, VALG, and HRA were evaluated for associations with the clinical variables using a linear mixed effect model, and the population mean of the conformational variable served as the reference point for the comparisons. To investigate associations between INC and clinical variables, a mixed cumulative logit model was used to model the probability of a higher INC grade with odds ratios (ORs) and their 95% confidence intervals (CIs) to quantify the effect. The models included breed and each clinical variable separately as fixed terms, and dog as the random subject effect for clinical variables not specified to one limb (weight, questionnaire, lameness in orthopedic examination, height, chest circumference, static weight distribution between front and hind legs). We decided to consider lameness as a clinical variable not specified to one limb because the movements of lame dogs were often abnormal in a complex way and only in a minority of cases could the dog be indicated to be lame in only one specific forelimb. The limb was included as an additional fixed term for clinical variables specified to one limb [pain, PROM, static weight distribution to each leg, variables of gait analysis, distance of the olecranon from the ground and its ratio with chest circumference (limb/chest ratio), elbow joint osteoarthritis, and CUCR]. The interaction between breed and each clinical variable was initially kept in all models but was removed from the final models where the interactions were statistically insignificant. However, each breed was assessed separately for the association between INC and limb/chest ratio as well as VALG and distance of the olecranon from the ground, as these associations were different from breed to breed.

*P*-values of < 0.05 were considered statistically significant. All statistical analyses were performed with SAS^®^ system for Windows, version 9.4 (SAS Institute Inc., Cary, NC, USA). All statistical tests were selected and completed by a biostatistician.

## 3. Results

### 3.1. Study population

Altogether 112 dogs of the three chondrodysplastic dog breeds were included in the study: 30 Standard Dachshunds (10 long haired, 10 smooth haired, and 10 wire haired), 29 Skye terriers, and 53 Glen of Imaal terriers. Thus, data from a total of 224 front limbs were available for the study. Incompatible temperament hindered passive range of motion measurements of one dog and gait analysis of another dog. Two dogs were excluded from static weight bearing measurement and gait analysis because of furunculosis. Two dogs were excluded from gait analysis due to a technical problem with the pressure-sensitive walkway at the time, and logistic issues prevented static weight bearing measurement in two dogs. One dog was excluded from the radiographic examination because of a heart murmur found in the clinical examination, a condition considered to be a risk for sedation. All of these dogs participated in other parts of the study. Two dogs had such severe ALD that CrCd radiographs could not be obtained. Eight images were excluded from the HRA measurement due to obliquity and summation of the thoracic soft tissues on the elbow joint - both of these issues were subjectively more common in the very short-limbed Dachshunds. A detailed description of the study population is presented in Table 2. After recruitment, one Glen of Imaal terrier reached 11 years of age (1 year above the inclusion age) by the time of the study appointment.

**Table 2:** Description of the study population for all dogs and by breed presented as mean ± SD (range).

**Breed**	***N***	**Sex**		**Age (years)**	**Weight (kg)**	**Height at olecranon (cm)**	**Height at withers (cm)**	**Chest circum-ference (cm)**	**Limb/chest ratio**
		**F**	**M**						
All dogs	112	61	51	3.6 ± 2.2 (1–11)	14.5 ± 4.5 (6.0–24.8)				
Dachshund	30	17	13	3.2 ± 1.3 (1–7)	9.2 ± 1.9 (6.0–12.8)	14 ± 2 (12–24)	25 ± 3 (22–36)	47 ± 4 (39–55)	0.30 ± 0.04 (0.25–0.46)
Skye terrier	29	15	14	3.4 ± 1.9 (1–8)	14.5 ± 2.4 (10.0–21.5)	16 ± 2 (15–25)	29 ± 1 (27–32)	51 ± 8 (15–61)	0.34 ± 0.14 (0.26–1.06)
Glen of Imaal terrier	53	29	24	4.0 ± 2.7 (1–11)	17.5 ± 3.6 (8.3–24.8)	18 ± 1 (15–22)	32 ± 3 (27–39)	58 ± 4 (46–69)	0.30 ± 0.03 (0.25–0.39)

N, number of dogs; F, female; M, male; SD, standard deviation.

### 3.2. Differences in front limb conformation between the breeds

The three studied breeds differed significantly in their front limb conformation, as breed was the largest explanatory factor for the differences in the measured values in all examinations. The detailed results are shown in Tables 3, 4. Significant differences existed between breeds in all variables, except mean VALG angles, which were similar between Glen of Imaal terriers and Dachshunds. No significant differences emerged between the left and right limb for any of the variables in any of the breeds. Additionally, when investigating whether conformational variables correlated with each other, only weak correlations (0.36 at the most) (25) were found.

**Table 3:** Front limb conformation described with ROT and VALG measured with a universal goniometer, and HRA measured from craniocaudal antebrachial radiographs presented as mean ± SD (range).

**Breed**	**Front limb conformational measurements**		
	**ROT**	**VALG**	**HRA**
Dachshund	20°± 7° (4°–35°)	23°± 12° (3°–57°)	78°± 8° (58°–96°)
Skye terrier	27°± 10° (−7° to 43°)	33°± 10° (11°–61°)	61°± 10° (33°–81°)
Glen of Imaal terrier	34°± 14° (0°–75°)	22°± 13° (5°–52°)	74°± 7° (53°–90°)

ROT, thoracic limb external rotation; VALG, carpal valgus; HRA, humeroradial angle; SD, standard deviation.

**Table 4:** Frequencies (*n*) and proportions (%) of INC grades, elbow joint osteoarthritis, and radiographic bone remodeling of cranial distal ulnar growth plate (CUCR) in the Dachshund, Skye terrier, and Glen of Imaal terrier.

**Breed**	**Total**	**Frequencies of radiographic findings**							
		**INC0**	**INC1**	**INC2**	**INC3**	**OA**	**CUCR0**	**CUCR1**	**CUCR2**
	***n***	***n* (%)**							
Dachshund	60	5 (8.3)	47 (78.3)	6 (10.0)	2 (3.3)	4 (6.7)	1 (1.7)	49 (81.7)	10 (17.7)
Skye terrier	54	2 (3.7)	31 (57.4)	17 (31.4)	4 (7.4)	19 (35.2)	1 (1.9)	22 (40.7)	31 (57.4)
Glen of Imaal terrier	106	29 (27.3)	72 (67.9)	5 (4.7)	0 (0.0)	0 (0.0)	20 (18.9)	81 (76.4)	5 (4.7)

N, number of dogs; INC, incongruity grade; OA, osteoarthritis; CUCR, cranial cortical ulnar remodeling.

### 3.3. Associations between conformation and clinical findings

When measurements of all dogs were pooled, significant associations between clinical findings (Tables 2–7) and ROT, VALG, HRA, and INC were detected (Table 8).

**Table 5:** Frequencies (*n*) and proportions (%) of dogs with pain and lameness noted in orthopedic examination and as reported by the owner of the Dachshund, Skye terrier, and Glen of Imaal terrier.

**Breed**	**Orthopedic examination**				**Questionnaire**	
	**Lameness**	**Pain**			**Lameness**	
		**Shoulder**	**Elbow**	**Carpus**	** < 1 year**	**>1 year**
	***n* (%)**					
Dachshund	2 (3.3)	16 (26.7)	8 (13.3)	0 (0.0)	7 (11.7)	5 (8.3)
Skye terrier	4 (6.9)	6 (10.3)	15 (25.9)	8 (13.8)	15 (25.9)	7 (12.0)
Glen of Imaal terrier	16 (15.1)	5 (4.7)	10 (9.4)	12 (11.3)	14 (13.2)	13 (12.2)

**Table 6:** Passive ranges of motion in degrees of the carpus, elbow, and shoulder joints in the Dachshund, Skye terrier, and Glen of Imaal terrier presented as mean ± SD (range).

**Breed**	**Ranges of motion**					
	**Carpus**		**Elbow**		**Shoulder**	
	**Flexion**	**Extension**	**Flexion**	**Extension**	**Flexion**	**Extension**
Dachshund	35°± 5° (25°–50°)	183°± 7° (170°–200°)	36°± 5° (30°–45°)	142°± 8° (125°–155°)	55°± 5° (40°–65°)	146 °± 8° (120°–160°)
Skye terrier	32°± 7° (20°–55°)	186°± 16° (155°–240°)	34°± 4° (25°–45°)	145°± 13° (110°–170°)	52°± 6° (40°–65°)	153°± 9° (130°–170°)
Glen of Imaal terrier	36°± 10° (20°–65°)	185°± 12° (160°–215°)	34°± 7° (20°–45°)	155°± 12° (120°–170°)	51°± 9° (25°–70°)	155°± 10° (120^°°^–170)

SD, standard deviation.

**Table 7:** Static weight bearing as a proportion (%) of weight distributed to a single front limb, single hind limb, and the front/hind ratio for static weight bearing and gait parameters of stance time (s), step length (cm), and total pressure in the Dachshund, Skye terrier, and Glen of Imaal terrier presented as mean ± SD (range) for static weight bearing measurements, and mean ± SD for gait analysis.

**Breed**	**Static weight bearing**						**Gait analysis**			
	**F (%)**	**Ftot (%)**	**H (%)**	**Htot (%)**	**F/H**	**Step length (cm)**	**Stance time (s)**		**Total pressure index**	
							**Front**	**Hind**	**Front**	**Hind**
Dachshund	33 ± 5 (23–45)	66 ± 4 (63–69)	17 ± 3 (12–25)	34 ± 4 (31–37)	2.0 ± 0.52 (1.0–2.9)	29 ± 3	0.12 ± 0.018	0.10 ± 0.0145	22 ± 4	13 ± 2
Skye terrier	33 ± 5 (24–42)	65 ± 7 (60–70)	17 ± 3 (12–25)	35 ± 7 (30–46)	2.0 ± 0.40 (1.1–2.8)	33 ± 3	0.15 ± 0.019	0.13 ± 0.017	32 ± 5	19 ± 3
Glen of Imaal terrier	33 ± 5 (22–48)	69 ± 2 (67–70)	17 ± 3 (11–23)	32 ± 2 (30–33)	2.0 ± 0.47 (1.0–3.4)	33 ± 4	0.16 ± 0.019	0.12 ± 0.0135	38 ± 8	26 ± 5

SD, standard deviation; F, weight distribution of single front limb; Ftot, total weight distribution of front limbs; H, weight distribution of single hind limb; Htot, total weight distribution of hind limbs; F/H, front to hind ratio of weight distribution.

**Table 8:** Statistically significant associations of conformational variables (VALG, ROT, INC, and HRA) with clinical and kinematic findings in Dachshunds, Skye terriers, and Glen of Imaal terriers.

**↑VALG**		**↓ROT**	
Limb/chest ratio↓	*p* = 0.036	Weight↑	*p* = 0.003
Front limb height (Glen)↓	*p* = 0.023	Height at withers (right)↑	*p* = 0.031
Chest circumference↑	*p* = 0.027	Limb/chest ratio↓	*p* < 0.001
Shoulder pain↑	*p* = 0.027	Chest circumference↑	*p* < 0.001
Lameness >1 year (right) ↑	*P* = 0.002	^*^Carpal pain↑	*p* = 0.076
Lameness >1 year (left)↑	*p* = 0.006	Lameness < 1 year (right)↑	*p* = 0.006
Carpal flexion↓	*p* = 0.001	Lameness >1 year (left)↑	*p* = 0.029
Elbow extension↓	*p* = 0.016	Carpal flexion↓	*p* = 0.016
*Shoulder extension↓	*p* = 0.098	Carpal extension↓	*p* < 0.001
		Total pressure front↑	*p* = 0.004
		Total pressure hind↑	*p* < 0.001
↑**INC**		↓**HRA**	
Limb/chest ratio (Glen)↓	*p* = 0.011	Limb/chest ratio↑	*p* = 0.003
Front limb height↓	*p* = 0.043	Front limb height↓	*p* = 0.002
Lameness (left)↑	*p* = 0.038	Chest circumference↓	*p* < 0.001
Shoulder flexion↓	*p* = 0.027	Lameness (left)↑	*p* = 0.026
Elbow joint osteoarthritis↑	p < 0.001	Elbow joint osteoarthritis↑	*p* = 0.001
CUCR2 vs. CUCR0↑	*p* = 0.037	Step length front↓	*p* = 0.021
CUCR2 vs. CUCR1 ↑	*p* = 0.044	Step length hind↓	*p* = 0.005

↑, increase in value; ↓, decrease in value; VALG, carpal valgus; ROT, thoracic limb external rotation; INC, elbow incongruity grade; HRA, humeroradial angle; CUCR, cranial ulnar cortical remodeling.

^*^Close to significance.

An increasing ROT was significantly associated with increasing weight, decreased carpal range of motion in both flexion and extension, and increased frequency of carpal pain. Dogs with more ROT had owner-reported lameness during growth and adulthood. They also had a smaller limb/chest ratio and smaller height at withers. In addition, they had a higher total pressure during trot in both front and hind legs.

The dogs with more VALG were significantly more likely to have shoulder pain as well as lameness during growth and adulthood as reported by the owner. Decreases in carpal flexion and elbow and shoulder extension ranges were also associated with increasing VALG. In addition, dogs with larger VALG had a smaller limb/chest ratio.

Decreasing HRA as a measure of radial subluxation was significantly associated with lameness, shorter front and hind limb step length, larger limb/chest ratio, and elbow joint osteoarthritis.

Dogs with higher INC grades had significantly more lameness (OR 3.25, CI 1.07–9.88), decreased shoulder flexion (OR of higher INC grade when flexion decreased 5° was 1.35, CI 1.04–1.77), more elbow joint osteoarthritis, and a smaller limb/chest ratio. CUCR1 and CUCR2 were seen more commonly with higher INC grades; OR for CUCR2 vs. CUCR1 was 3.22 (CI 1.03–10.01) and CUCR2 vs. CUCR0 was 5.77 (CI 1.12–29.80). No significant difference emerged between CUCR0 and CUCR1.

### 3.4. Breed-specific observations

#### 3.4.1. Dachshunds

The Dachshund was the smallest of the chondrodysplastic breeds (Table 2). They had the lowest prevalence of lameness noted in the orthopedic examination (3.3%) (Table 5) but a high frequency of shoulder pain (26.7%) and a slightly smaller maximal shoulder extension (146° SD ± 8°) than the other two breeds (Table 6). Dachshunds had the smallest mean ROT (20° ± 7°) of the front limb. They also had the largest mean HRA (78° ± 8°) in radiographs, which indicates less elbow subluxation than in the other breeds (Table 3). The majority (78.3%) of Dachshunds were graded INC1, and elbow joint osteoarthritis was not a frequent finding (6.7%) (Table 4). CUCR1 was found in the majority (81.7%) of Dachshunds (Table 4).

#### 3.4.2. Skye terriers

The Skye terrier owners commonly observed front limb lameness during growth (25.9%). In the orthopedic examination, pain was most commonly noted in the elbow joint (31%) (Table 5). The Skye terriers had the largest mean VALG (±SD) of 33° (±10°), with a maximum of 61°. The smallest mean HRA of 61° (±10°) was observed in Skye terriers, with a minimum of 33° signifying marked lateral radial subluxation (Table 3). The breed had the largest prevalence of high INC grades, with INC2 found in 31.4% and INC3 in 7.4% of dogs (Table 4). CUCR2 was common (57.4%), which was the highest prevalence of this grade across the three breeds (Table 4). Elbow joint osteoarthritis was also most prevalent in the Skye terrier (35.2%).

#### 3.4.3. Glen of Imaal terriers

The Glen of Imaal terrier was the largest of the breeds (Table 2). Lameness was detected in the orthopedic examination more frequently (15%) than in the other breeds, and the most common site of pain was the carpal joint (11.3%) (Table 5). The least carpal flexion was seen in this breed (36° ± 10°). On the other hand, elbow joint maximal passive extension (155° ± 12°) was slightly higher than in the other breeds (Table 6). The Glen of Imaal terrier had the largest mean front limb ROT at 34° (±14°), with the maximum value being 75°, signifying marked external rotation (Table 3). The highest frequency of INC0 was found in this breed, with a prevalence of 27.3%, while INC2 was present in 4.7% and INC3 was not observed in any of the Glen of Imaal terriers (Table 4). The Glen of Imaal terriers had the highest frequency of CUCR0 (19%) and the lowest frequency of CUCR2 (5%) (Table 4).

## 4. Discussion

### 4.1. Limb conformation

Our findings regarding front limb conformation in the three chondrodysplastic breeds confirm that the consequences of the supposed premature closure of the distal ulnar growth plate manifests differently in different breeds, with varying patterns of clinical, radiological, and biomechanical findings. This is not surprising considering that the breed standards, which direct the breeding choices, describe the front limb conformation quite differently in each of these three breeds. The breed standard for the Dachshund defines the desired appearance as “upper arms close fitting to ribs but free in movement, elbows turning neither in nor out, forearm as straight as possible, carpal joints slightly closer together than the shoulder joints” (26). The Skye terrier breed standard defines front limbs as “legs short and muscular, forefeet pointing truly forward” (27). And, finally, the Glen of Imaal terrier is desired to present with “forelegs short, bowed, and well boned, front feet to turn out slightly from pasterns” (28). Thus, Dachshunds should have as straight forearms as possible, Skye terriers short forearms with cranially pointing paws, and Glen of Imaal terriers short and bowed forelimbs with mild outward rotation of the distal limb. In our study population, some of the conformational features were exaggerated in relation to the descriptions of the front limbs in the breed standards. For example, the Glen of Imaal terriers often had an external rotation that could not be described as mild, with a maximum rotation angle of 75° and a mean of 34°, which is markedly more than, for example, the mean rotation angle of 20° in Dachshunds. Moreover, Skye terriers often had marked proximal lateral radial subluxation, as the HRA was small. Since the dogs were enrolled by owner preference, caution is warranted in drawing conclusions regarding the breeds as a whole.

### 4.2. Clinical significance

The findings of our study highlight that an exaggerated interpretation of breed standards can have a detrimental impact on the welfare of dogs at least in these three breeds. Dogs with more severe INC were the ones with shorter front limbs. They had less flexion in the shoulder joint, were more often lame in orthopedic examination, and had an increased risk for elbow joint osteoarthritis. The consequences of elbow incongruity on the welfare of the dog are well-understood. INC was not associated with pain in palpation, which is in accordance with previous studies. For example, weight bearing-induced pain during locomotion may not be provoked in the orthopedic examination. Joint palpation in lateral recumbency does not necessarily cause a pain response, but significant lameness or shifting of weight off the limb may be seen when the limb is loaded (7, 13, 29, 30).

Increased front limb external rotation and carpal valgus were associated with a decreased range of motion of the carpal joint and decreased extension of the elbow and shoulder joints across all breeds. From a functional anatomy point of view, this is logical, although not reported previously. To our knowledge, there are no earlier publications on PROM of chondrodysplastic breeds. Compared with non-chondrodysplastic dogs, the mean carpal extension angle was smaller in our study (185°) than the carpal extension of 192° recorded in Labrador retrievers (19). The carpal flexion angle in our study (35°) was close to the value in Labrador retrievers (32°) (19). Moreover, the elbow joint extension in chondrodysplastic dogs was markedly less in our population (147°) than in non-chondrodysplastic breeds (Labrador retrievers 165°) (19). Again, the flexion of the elbow was similar in our study population (34–36°) and in Labrador retrievers (36°). In all of the three breeds in our study, extension of the shoulder joint was less (mean for the three breeds 151°) than in previously published values in Labrador retrievers (165°) (19). The ALD present to a various extent in chondrodysplastic dogs might explain the difference in the passive ranges of motion in our study and those reported for non-chondrodysplastic dogs.

Although the weight distribution was not associated with any of the clinical variables, we found that weight was mainly distributed to the front limbs (65–69%, Table 7). This is slightly more (63%) than what has recently been reported for chondrodystrophic dogs using digital bathroom scales (31). The difference between studies could be explained by different breeds and different measurement methods. Weight distribution of the dogs in our study is comparable to that in English Bulldogs, which have been reported to have 67.3% of total weight distributed to their front limbs, as measured with a pressure-sensitive platform (13), as used also in our study. In gait analysis, the total pressure was significantly higher in both front and hind limbs of the dogs with more ROT, which is probably due to these dogs being heavier than the dogs with less ROT. A lateral swinging motion of the front limb during swing phase has been described in association with ALD and could serve as one explanation for our finding (4). For example, if a dog with more external rotation needs to make this type of lateral swinging motion to gain clearance from the ground, the lateral motion of the limb could require the dog to lean more on the supporting leg to balance the motion (4). Changes in the way the dog uses its limbs can cause changes in the mechanical stresses in bones, joints, ligaments, tendons, and muscles, as well as in skin and soft tissues of the paw if paw position is altered. An abnormal loading of the joint can result in wearing of the joint surface, which can lead to osteoarthritis (32–34). A limitation of gait analysis possibly affecting the results is that we did not control trotting speed of dogs, and speed was not made proportional to the weight of the dog. However, as some of the dogs affected by ALD could have marked variation or difficulties in locomotion, we decided to allow the dogs to trot at their preferred speed. To ensure measurement consistency in individual dogs, several passes were performed to collect a minimum of 20 gait cycles for analysis for each dog (35, 36).

Interestingly, more than 25% of Dachshunds had shoulder pain in the orthopedic examination, in addition to less PROM in the shoulder joints than the other two breeds. Dysplasia of the shoulder joint could be a reason for the pain and less PROM (37). Literature on how chondrodysplasia affects the shoulder joint is scarce. However, this is probably another less acknowledged feature of the chondrodysplastic phenotype, as the *FGF4* retrogene of chromosome 18 has a mild inhibitory effect also on the growth of the scapula (3). Similarly, as in more distal parts of the front limb, this phenotype probably differs between chondrodysplastic breeds.

Interestingly, the limb/chest ratio was found to be associated with all of our conformational variables. Our findings imply that short limbs in a larger and more robust dog with a larger chest is a combination prone to conformational abnormalities of the front limbs. However, dogs with a smaller HRA (more lateral elbow subluxation) had a larger limb/chest ratio, even though they had a smaller chest. In these dogs, the large limb/chest ratio was attributable to significantly shorter front limbs. According to a recent study investigating the effects of *FGF4* retrogenes on the conformation, chondrodysplasia itself has no impact on chest height or width at least in Alpine Dachsbracke and Schweizer Niederlaufhund (3), suggesting that other, yet unknown genes affect the growth of the chest.

### 4.3. Novel methods for measuring radiographic front limb conformation

#### 4.3.1. Humeroradial angle

Lateral radial subluxation is a common consequence of ulnar growth disturbances, such as ALD and exaggerated chondrodysplastic conformation, but evaluation of its existence and quantity has thus far been based merely on subjective assessment (4, 5, 7, 30). Lateral radial subluxation has been reported in up to 93% of the more complex biapical ALDs, which are common in chondrodysplastic breeds (5, 30). We developed the HRA measurement to quantify lateral radial subluxation in the elbow joint. The HRA shares some resemblance with the center of rotation of angulation (CORA) methodology, which was used in a recent study to evaluate the center and amount of radial curvature in ALD of different chondrodysplastic breeds (38). However, CORA and HRA measure different aspects of the forearm conformation. With CORA, the shape of the radius can be defined in detail, but it does not take into account lateral radial subluxation, which can be assessed with HRA. On the other hand, HRA does not evaluate the curvature of the radius. Thus, these two methods are recommended for use together.

In our study, the HRA differed substantially between the breeds, as the difference of the mean HRA between Skye terriers and Dachshunds was 17°, with Skye terriers having the smallest HRA (most lateral subluxation). Variation between individual dogs in all breeds was also large (range 33°-96°). A decreasing HRA (increasing subluxation) was significantly associated with shorter front limbs, shorter step length, pain, and elbow joint osteoarthritis, indicating that lower HRA values have an impact on chondrodysplastic dogs' welfare. One limitation in this assessment is that sometimes the very short and curved front limb and its intimate proximity to the soft tissues of the upper limb and thoracic wall may hinder acquisition of a radiograph suitable for measurements in this projection. In our study, 10 of the 224 radiographs could not be measured for HRA.

As HRA is simple and quick to measure from a CrCd radiograph, it could be an additional projection along with an ML projection in radiographic screening protocols. However, to get accurate measurements, careful positioning of the often very short and curved front limb is mandatory. Intra- and inter-evaluator reliability should also be tested, as the measurements here were made only once by one assessor. Our results support validation of the proposed method, as the measurements appeared to have a clinical impact. Further studies are needed also to define reference values for HRA angles that could be considered acceptable for chondrodysplastic breeds.

#### 4.3.2. Cranial ulnar cortical remodeling

**Figure 3:**
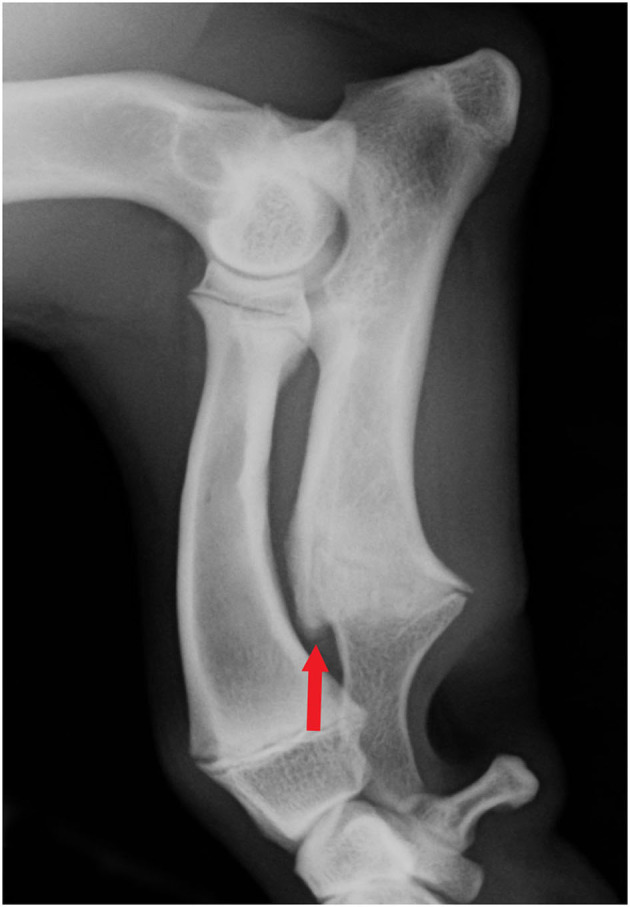
Mediolateral antebrachial radiograph of a young Skye terrier (not from the study group) with visible open growth physis. The remodeling of the cranial metaphysis and physis is clearly visible (arrow).

Cranial cortical bone remodeling is frequently seen in ML radiographs of chondrodysplastic dogs. We identified a distinct bulge (CUCR2) at the site of the distal ulnar growth plate that was found to be associated with the conformational variables of ALD in our study. However, this interosseous space can also have other changes in the cortical bone (CUCR1), for example, as a result of ossification of the interosseous ligament of the antebrachium, which were not found to be associated with the conformational variables that we studied. Thus, the distinct ossified bulge on the cranial aspect of the distal ulnar growth plate should be considered an independent entity, and it appears to be a consequence of abnormal ulnar growth (Figure 3). This often prominent phenomenon is explicitly mentioned only once in the literature by Lau (4), who referred to it as “distal synostosis”.

We introduce a simple subjective grading protocol for assessing the severity of bone remodeling at this site. The mild form (CUCR1) was common in both Dachshunds and Glen of Imaal terriers, whereas most of the more prominent (CUCR2) findings were in Skye terriers. This bony bulge is probably caused by a disturbance in the ossifying process of the distal ulnar growth plate, and it might correlate with the timing and severity of the premature closure of the growth plate. CUCR was associated with INC, as CUCR2 was significantly more common with higher INC grades. It can be speculated that the earlier the ulnar growth terminates, the larger the bulge and the more severe the incongruity (39, 40). Further longitudinal and reliability studies on growing chondrodysplastic dogs would yield more insights into the phenomenon of premature closure of the distal ulnar growth plate.

### 4.4. Relevance to breeding

As the premature closure of the distal ulnar growth plate and the consequent ALD are suspected to be hereditary in chondrodysplastic dogs (4), appropriate screening schemes should be utilized to prevent this debilitating condition. An interesting and important finding of the present study was that ROT, VALG, and HRA occurred as independent findings. This emphasizes that a growth disorder can cause a combination of conformational changes that can occur independently of each other, and more than one method for assessing the front limb conformation is needed when selecting dogs for breeding. For example, the Glen of Imaal terriers in our study often had congruent elbow joints, but at the same time had severe carpal valgus with carpal pain, highlighting the usefulness of measuring the external rotation as a part of the evaluation at least in this breed. In Finland, a protocol for radiographic grading of elbow incongruity in chondrodysplastic dogs (6, 16) was introduced some years ago (41), but adding ROT, VALG, and HRA measurements to the screening protocol would probably increase the impact of screening in selected breeds.

## 5. Conclusions

INC, ROT, VALG, and HRA are each associated with several measurable clinical abnormalities in limb function, and thus, may have effects on dogs' quality of life due to the related pain and functional limitations. Recognition of these abnormalities is therefore warranted. Our findings suggest that assessing for VALG, ROT, and HRA could be valuable supplementary methods to radiographic INC grading when screening dogs for breeding. This would increase the sensitivity of detecting an abnormal front limb structure to allow selection of healthier conformation of front limbs for breeding. For selective purposes, reference values should be set in future studies. Also, breed standards of chondrodysplastic breeds should be reviewed and the descriptions of unhealthy limb conformation revised.

## Data availability statement

The raw data supporting the conclusions of this article will be made available by the authors, without undue reservation.

## Ethics statement

The animal study was reviewed and approved by National Animal Experiment Board in Finland (ESAVI/9184/04.10.07/2014) and the University of Helsinki Viikki Campus Research Ethics Committee (Statement 2/2017). Written informed consent was obtained from the owners for the participation of their animals in this study.

## Author contributions

AL and HP: conception, design, and drafting the article. HP and HH: acquisition of data. HP, AL, JJ, and HH: analysis and interpretation of data. AL, HP, HH, JJ, and OL-V: revising article for intellectual content. All authors contributed to the article and approved the submitted version.
